# Biological Importance of Cotton By-Products Relative to Chemical Constituents of the Cotton Plant

**DOI:** 10.3390/molecules22010093

**Published:** 2017-01-06

**Authors:** Mary A. Egbuta, Shane McIntosh, Daniel L. E. Waters, Tony Vancov, Lei Liu

**Affiliations:** 1 Southern Cross Plant Science, Southern Cross University, Lismore, NSW 2480, Australia; m.egbuta.10@student.scu.edu.au (M.A.E.); daniel.waters@scu.edu.au (D.L.E.W.); 2 NSW Department of Primary Industries, Wollongbar Primary Industries Institute, NSW 2477, Australia; shane.mcintosh@dpi.nsw.gov.au (S.M.); tony.vancov@dpi.nsw.gov.au (T.V.)

**Keywords:** cotton gin trash, cotton residues, *Gossypium*, terpenoids, phenolics, phytochemicals

## Abstract

Although cultivated for over 7000 years, mainly for production of cotton fibre, the cotton plant has not been fully explored for potential uses of its other parts. Despite cotton containing many important chemical compounds, limited understanding of its phytochemical composition still exists. In order to add value to waste products of the cotton industry, such as cotton gin trash, this review focuses on phytochemicals associated with different parts of cotton plants and their biological activities. Three major classes of compounds and some primary metabolites have been previously identified in the plant. Among these compounds, most terpenoids and their derivatives (51), fatty acids (four), and phenolics (six), were found in the leaves, bolls, stalks, and stems. Biological activities, such as anti-microbial and anti-inflammatory activities, are associated with some of these phytochemicals. For example, β-bisabolol, a sesquiterpenoid enriched in the flowers of cotton plants, may have anti-inflammatory product application. Considering the abundance of biologically active compounds in the cotton plant, there is scope to develop a novel process within the current cotton fibre production system to separate these valuable phytochemicals, developing them into potentially high-value products. This scenario may present the cotton processing industry with an innovative pathway towards a waste-to-profit solution.

## 1. Introduction

Cotton (*Gossypium*) is naturally a perennial plant that is now commercially cultivated as an annual plant in many parts of the world [1]. The cotton bud is the most utilized part of the plant and is the starting raw material for a wide range of products, such as textiles, edible oil, paper, livestock feed, and medicinal products, to name a few [2,3,4,5,6,7]. Cotton fibre has many positive characteristics (comfort, colour retention, absorbency, strength) [2] and, hence, global cultivation has increased to an estimated production of over 23 million tonnes in 2013–2014 [8]. This increase in cotton production has resulted in tonnes of waste remaining after harvesting and processing (ginning), which has contributed to a growing challenge of its disposal [9,10].

Non-cotton fibre biomass residues generated from cotton production and processing includes cotton gin trash (CGT), post-harvest field thrash (PHT), and crushed seeds from which oil has been extracted. Post-harvest trash (PHT) are the remaining parts of the plant left on the field, while CGT is centralised at gins and is comprised mainly of sticks, burrs (calyx), leaves, and soil [9,11]. These by-products of the cotton industry, although underutilized, are being used as soil composts and cottonseed meal nutritional supplements for livestock feed [10,12,13,14]. Other methods of utilization of cotton industry by-products have been explored, particularly as a low-cost biomass feedstock for commercial bioenergy/biofuel applications [15,16,17], mostly from CGT.

The entire cotton plant has the potential to be a source of valuable compounds, such as terpenes, phenolics, fatty acids, lipids, carbohydrates, and proteins [18,19,20,21,22,23]. These compounds, which are distributed in seeds, bolls, calyx, leaves, stalks, stems, and roots of the plant [20,23,24,25] play functional biological roles in humans and animals [21,26,27,28,29]. Gossypol, a poly-phenolic with potential contraceptive effects [30] and trans-caryophyllene, a terpenoid having anti-inflammatory and cytotoxic properties [31,32], are examples of compounds present in cotton with potential beneficial impact on humans and animals. Thus, by-products generated by the cotton industry (CGT, PHT, and crushed seeds) may represent a potential source of valuable extractives due to the distribution of chemical compounds throughout the whole cotton plant. 

Agricultural products and by-products other than cotton are being exploited for generation of energy and materials [33], serving as a means of recycling and reducing organic wastes in the environment. Biomass from soybean, rice, and sugar cane are examples of agricultural by-products currently utilized for these purposes [34,35,36,37]. Valuable chemical extractives may also occur in some of this agricultural biomass [38], although these potential resources have not been fully exploited. 

The multitude of potentially valuable chemicals that could be derived from cotton production by-products has gained little attention. Hence, exploiting the full potential of cotton waste will be beneficial to both the cotton industry and the local environment. A more detailed investigation is necessary to provide answers to such questions as: what is the nature of the extractives present in these by-products; how do the chemical profiles vary between cotton species and/or varieties and what potential uses do the waste products have as a source of high value compounds. To provide answers to some of these questions, this review begins with an overview of cotton production, the source of industry wastes, and its current utility. This review continues with a further discussion of current knowledge of chemical extractives present in the cotton plant and the distribution of these compounds within the plant highlighting their potential phytochemical properties which may be of value to both the pharmaceutical and agricultural sector. 

## 2. Cotton

Cotton, the *Gossypium* genus in the tribe *Gossypiae*, in the family Malvaceae, can be generally divided into two types: cultivated and wild cotton. Of 50 known species, only four (4) are cultivated, with the remaining 46 growing wild in the tropics and sub-tropics [39,40]. The four common cultivated cotton species are *G. hirsutum*, *G. herbaceum*, *G. barbadense*, and *G. arboreum*. These species vary in terms of fibre quality [41] defined by length, maturity, strength, and micronaire (cell wall thickness) of the fibre. The differences in fibre quality, yield, and adaptation to certain climatic conditions, has contributed to the preference of some cotton species over others [39]. All four cultivated cotton species are used for other purposes, including food production and medicinal application [21,42,43,44].

*G. hirsutum*, sometimes referred to as “upland, American or Mexican cotton”, is the most commonly cultivated of all cotton species [41]. *G. hirsutum*, is widely grown in its transgenic form because of its high yield and adaptability to different environmental conditions [45], although high temperatures can result in sterility and boll shedding [46,47]. The other species are predominant in parts of Asia and Africa and are seldom cultivated outside these regions [48,49], due to their inability to adapt to different climatic conditions [46] and poor yields [45,49]. *G. hirsutum* has been manipulated to improve yield and there has been some effort made toward enhancing the other cultivated species using transgenic approaches [50,51]. 

### 2.1. Transgenic Cotton

Fungal infection, pests, weed infestation and unfavourable environmental conditions are some of the challenges which result in poor yields [52,53,54,55,56,57]. These challenges prompted transgenic manipulation of cotton plants, to equip them with the ability to withstand unfavourable conditions and promote higher yields [58,59,60]. Bt cotton carries transgenes derived from the soil dwelling bacteria *Bacillus thuringiensis*, hence the name Bt cotton. Bt cotton cultivars carry the *Cry1Ac* gene making it resistant to the tobacco bud worm [61], or a combination of *Cry1Ac* and *Cry2Ab* gene, making it resistant to a wider range of cotton pests [62]. A new type of Bt cotton, which is yet to be introduced in the market, contains *Cry1Ac*, *Cry2Ab*, and *Vip3A* genes, with the last gene promoting increased resistance to more challenging pests such as lepidopteran insects [63,64]. Roundup Ready cotton, another transgenic type, contains a gene that confers resistance to glyphosate herbicide, enabling weed control without destroying the cotton. 

There have been other attempts to move favourable genes between species to enhance tolerance under unfavourable environmental conditions. For example, a heat shock protein gene GHSP26, thought to be responsible for drought tolerance in *G. arboretum*, has been transferred to *G. hirsutum*, thereby enhancing its ability to withstand drought [65].

## 3. Cotton Industry and Processing

**Figure 1:**
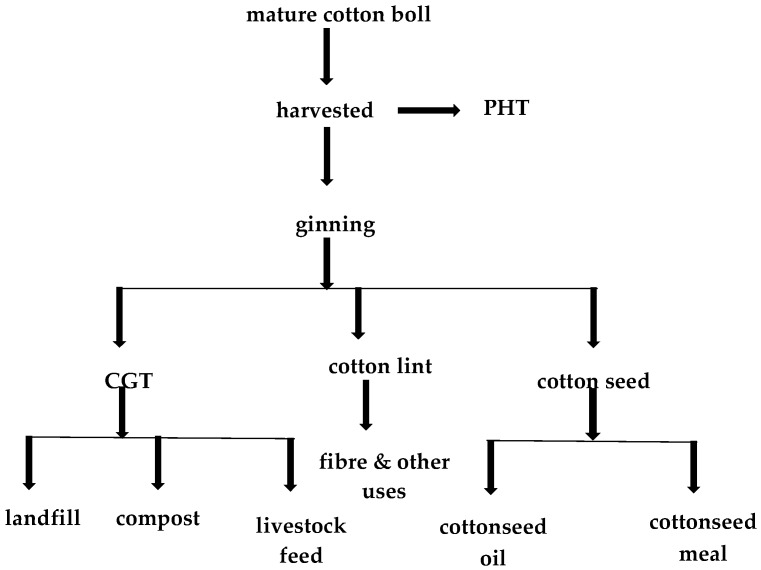
Flowchart of cotton processing from field to cotton gin products.

When fully matured, cotton bolls are picked and transported for processing, leaving the remaining plant as field trash. During the refining process or ginning of the harvested cotton, impurities are removed from the cotton fibres and are recovered as a processing by-product (CGT). Moreover, cotton seed is also processed to recover cotton seed oils and cotton seed meals. Cotton production generates three categories of waste products: (i) field trash (stems, flowers, leaves, and stalks); (ii) CGT (leaves, fibre, flowers, immature seeds, sticks and soil) [9] and cotton seed meal (from which oil has been extracted) (Figure 1).

### 3.1. Cotton Waste

Cotton by-products have provided producers with additional value, mainly in the form of livestock feed supplements and soil amendments [6,66]. Despite its abundance, field trash is generally viewed as having little value-added potential and is, therefore, not a resource that is utilised in standard farming practices. Field trash is currently slashed and left in the field where it provides some benefits through improving soil carbon and reducing soil erosion. The high cost associated with harvesting field trash for other uses is considered a major economic hurdle. 

Cotton seeds constitute 55% of the total ginned cotton by weight, whereas cotton fibre and CGT make up about 35%–40% and 10% respectively [67]. Although historically viewed as a waste by-product of cotton processing, cotton seed is now considered a high value co-product and an important part of the cotton processing value chain. Cotton seed is fractionated into high value oils and high protein meals, both with applications in food and feed industries. In contrast, CGT is considered a lower value waste with little value adding potential and the management of CGT is regarded as a financial burden to most ginning operations. CGT is generally disposed of in one of four ways: as solid waste (landfilling), composting and land application, incineration and, to a lesser extent, fed to livestock as a supplement [9,10]. Moreover, disposal options are tightly regulated by local environmental laws which add further restrictions.

CGT has a reasonable nutritional profile composed of dry matter (90%), crude protein (12%), total digestible nutrients (47%), calcium (11%), sodium (121 ppm), and iron (963 ppm) [66], and has been proven to contribute to the wellbeing of livestock [6,12]. Despite this, its use has been discouraged (and banned altogether in some countries) owing to the presence of residual chemicals which are used during cultivation [68]. This has resulted in CGT being widely used as fertilizer supplements [69,70] and composts to maintain/conserve soil moisture and composition that improve crop production [71,72].

Waste generated from cotton harvesting and cotton ginning mills are used as replacement components for inorganic-based filler materials and additives, for the production of thermoplastic composites poly(lactic acid) (PLA) and low-density polyethylene (LDPE) [73]. By-products from the cotton industry have also been processed to produce fulvic acid and silica [74]. Cotton trash has been investigated in numerous studies as a renewable feedstock in bioethanol production [15,16,75,76]. CGT is well suited as a biofuel feedstock because its composition has the attribute of high polysaccharide content (up to 50%) for effective and scalable conversion to biofuels.

A promising, yet less well documented use of CGT is in the manufacturing of biologically active compounds. There are a variety of chemical compounds which occur naturally in cotton plants with wide ranging activities. Given that such compounds are present in the cotton plant, it is plausible that the remaining trash also contains a proportion of biologically active molecules. These compounds and their uses are examined and discussed in the following sections of this review.

## 4. Chemical Compounds in Cotton

**Figure 2:**
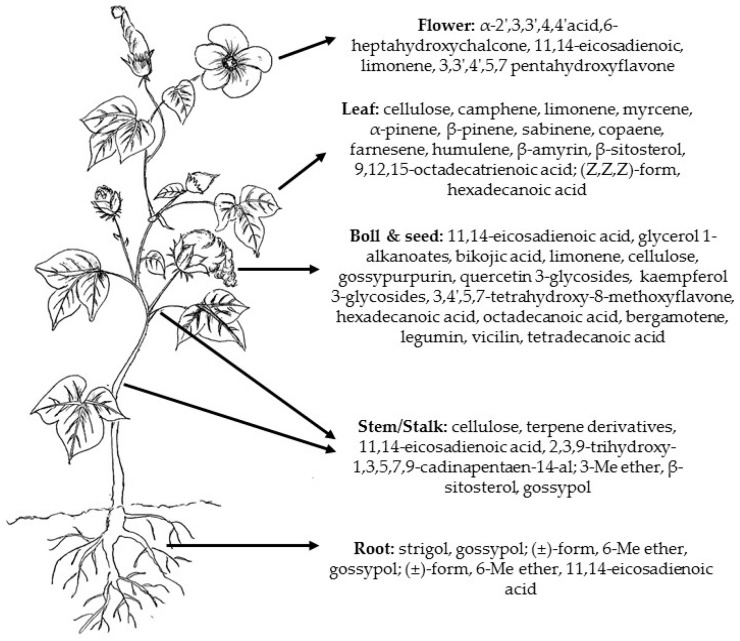
Distribution of common secondary metabolites in cotton plant.

Different compounds present in cotton play important roles during metabolism or interaction with the environment. Naturally-occurring compounds in cotton include terpenes, phenols, proteins, carbohydrates, fatty acids, and lipids [19] (Table 1). As with most plants, the distribution of these compounds vary between different parts of the cotton plant with some compounds concentrated in specific parts of the plant [77] (Figure 2). The distribution of these chemical compounds is related to their different properties and functionality in the plant. The various compounds found in cotton plant will be discussed, highlighting the chemistry, as well as their distribution within the plant.

### 4.1. Terpenes

**Figure 3:**
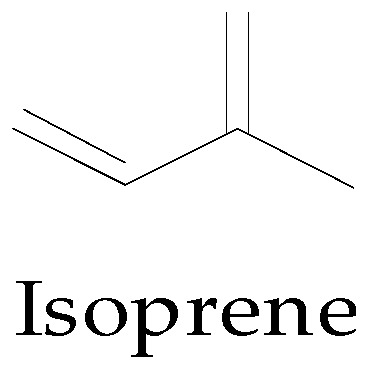
Chemical structure of isoprene (building block of terpenes).

Like most plants, the cotton plant is susceptible to insect, herbivore, and pathogen attack. In a bid to ward off these predators, compounds are produced by the plant as a defence mechanism. Terpenes are an important class of defence compounds synthesized in the cotton plant and are also the largest group of plant defence compounds [124,125]. They are major constituents of essential oils found in most plants and, as such, have been applied in the food, chemical, and cosmetic industry [126]. Terpenes are composed of units of a five-carbon compound, isoprene (Figure 3), linked together in a head to tail fashion [127], forming long chains or rings. They are classified into seven classes by the number of isoprene units they contain and include hemiterpenes, monoterpenes, sesquiterpenes, diterpenes, triterpenes, tetraterpenes, and polyterpenes [125,127,128,129]. Generally, hemiterpenes do not occur as free compounds but are bound to other non-terpene compounds [126], while terpenes modified by oxidation or a re-arrangement of the carbon skeleton are referred to as terpenoids.

According to Pare and Tumlinson [130] and Rose and Tumlinson [131], terpenes in cotton can be divided into two groups. The first are constitutive compounds that are present in the storage compartments of the cotton plant and are released immediately after insect feeding or damage. Some of these terpenes include α-pinene, β-pinene, limonene, caryophyllene, α-humulene, and myrcene. The second group of terpenes are referred to as inducible compounds which are synthesized de novo several hours after exposure to pests and herbivores and include β-ocimene, α-farnesene, β-farnesene, and linalool. Some of these terpenes occur in their enantiomeric forms in the plant with a reported occurrence of the negative forms e.g., -α-farnesene, -β-farnesene and -β-ocimene [131,132]. Monoterpenes, sesquiterpenes, triterpenes, and terpene derivatives mostly occur in the cotton plant, with monoterpenes, sesquiterpenes, and their derivatives being the most common [133]. The total concentration of terpenes in cotton plant is unclear, although accumulation of terpenes in cotton plant parts varies with up to 15.5 mg terpenoids reportedly accrued per fresh weight of cotton leaves [124]; 2.81 mg and 2.49 mg per foliage weight reported for monoterpenes and sesquiterpenes, respectively.

#### 4.1.1. Terpene Biosynthesis

Terpenes are synthesized via the acetate/mevalonate pathway [133] and mevalonate independent pathway [127,128]. The non-mevalonate pathway is also referred to as the deoxyxylulose phosphate (DXP) pathway or the methyl erythritol phosphate (MEP) pathway. Although terpene synthesis begins with photosynthesis, most studies identify the combination of three acetyl CoA molecules as the starting point of terpene or terpenoid biosynthesis via the acetate/mevalonate pathway [134]. 

**Figure 4:**
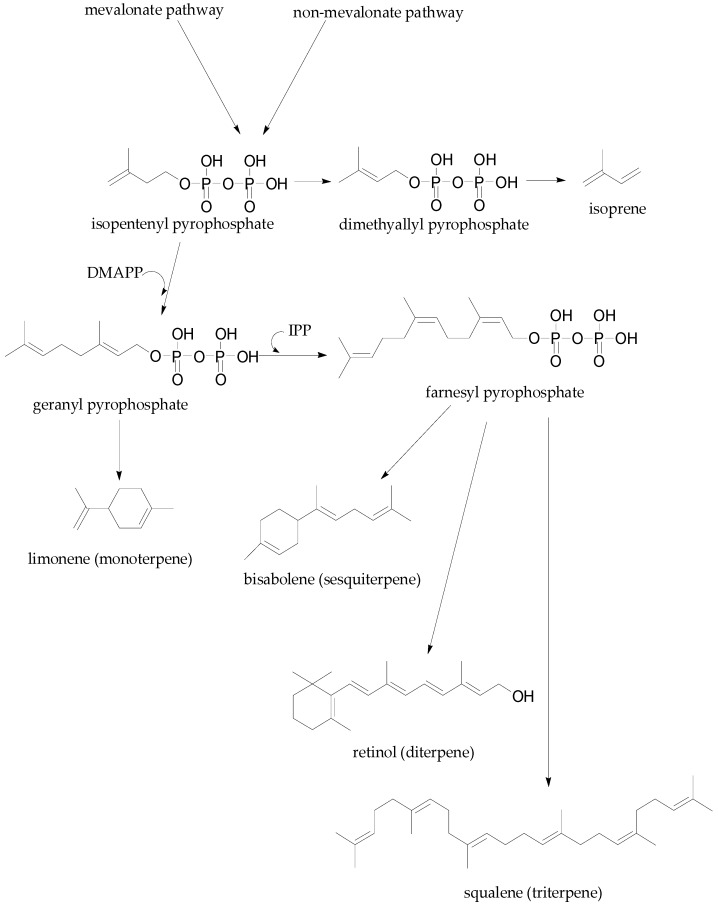
Biosynthesis of terpenes from isopentenyl pyrophosphate, a product of the mevalonate and non-mevalonate pathway.

The mevalonate and non-mevalonate pathway result in the formation of isopentenyl pyrophosphate (IPP) and dimethyl allyl pyrophosphate (DMAPP) which forms isoprene catalysed by isoprene synthase. Monoterpenes are synthesized in the plastids of plant cells from geranyl pyrophosphate (GPP) (Figure 4) which is formed from the combination of DMAPP and IPP catalysed by isoprenyl diphosphate synthases [135]. Sesquiterpenes (Figure 4) are synthesized in the cytosol from farnesyl pyrophosphate (FPP), which is formed from one molecule of GPP and IPP joined in a head to tail combination. The activity of sesquiterpene synthase enzymes converts FPP to sesquiterpenes via ionization reactions [136]. Other terpenes, diterpenes, and triterpenes are synthesized from FPP via the formation of geranyl geranyl diphosphate (GGDP) and squalene, respectively (Figure 4). Both pathways of terpene biosynthesis can, thus, be summarised into a four step process: (1) synthesis of IPP and isomerization to DMAPP; (2) addition of more IPP compounds; (3) terpene backbone formation by terpene synthase activity; and (4) enzymatic modification to induce specific functions of the terpenes [129].

#### 4.1.2. Monoterpenes (C10)

**Figure 5:**
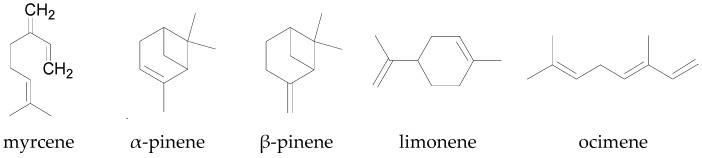
Chemical structures of some monoterpenes in plants, including cotton.

The monoterpenes (C_10_H_16_) are a class of terpenes that consist of two isoprene units and can be linear (acyclic), monocyclic (containing one ring), or bicyclic (containing two rings) [137]. There are over 1000 monoterpenes known to occur in nature and examples of common monoterpenes in plants include myrcene (acyclic), limonene (monocyclic), and pinene (bicyclic) (Figure 5) [137]. Together with the sesquiterpenes, monoterpenes are major constituents of essential oils extracted from various plant materials [138]. Biochemical modifications of monoterpenes such as oxidation, hydroxylation and rearrangement of atoms result in the formation of monoterpenoids such as geraniol and linalool [135,139]. In the cotton plant, there are some acyclic monoterpenes which belong to the group of constitutive compounds, such as α-pinene, β-pinene, and limonene amongst others, as well as herbivore-induced monoterpenes [130,132]. Although monoterpenes found in cotton are distributed in different parts of the plant, including leaves, seeds, flowers, stems, and roots, they are predominantly concentrated within the leaves and flowers (Figure 2) [23,124,140,141].

#### 4.1.3. Sesquiterpenes (C_15_)

**Figure 6:**
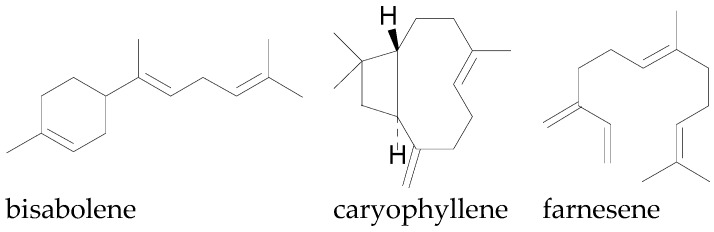
Chemical structures of some sesquiterpenes in cotton.

**Figure 7:**
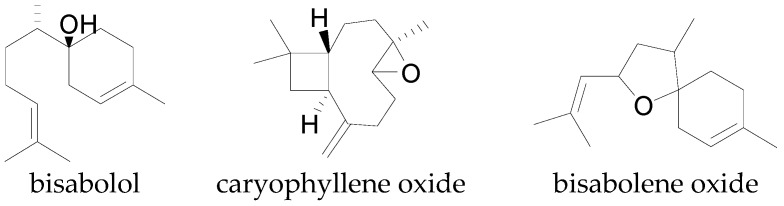
Some sesquiterpenoids isolated from cotton.

Sesquiterpenes (C_15_H_24_) are composed of three isoprene units either in acyclic or cyclic form and occur in most plants. Sesquiterpenes are not limited to higher plants; they have been discovered in micro-organisms, such as bacteria, fungi, and marine organisms [135]. The sesquiterpenes occur in many cotton species and have been extracted from the leaves, flowers, seeds, and bolls of cotton plants [21,142]. Bell [19] reported the total concentration of some sesquiterpenes in essential oil extracted from whole cotton plants up to 26.12% and 30.1% for *G. hirsutum* and *G. barbadense* respectively. The sesquiterpenes, α-bergamotene, caryophyllene, bisabolene, farnesene, humulene and copanene are some of the sesquiterpenes commonly associated with cotton (Figure 6), while oxidized forms such as bisabolol, bisabolene oxide, caryophyllene oxide, and other sesquiterpenoids also occur in the cotton plant [19] (Figure 7).

#### 4.1.4. Triterpenes (C_30_)

**Figure 8:**
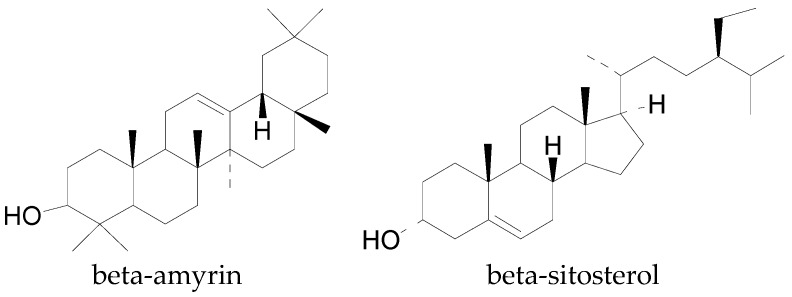
Triterpene derivatives in cotton.

There are no reports of diterpenes, tetraterpenes, and polyterpenes in cotton plants, however, two triterpene derivatives, β-sitosterol and β-amyrin montanate (Figure 8), were reported to occur in cotton leaves by Shakhidoyatov et al. [18]. Triterpenes are generally made of six (6) isoprene units and contain 30 carbon atoms with a molecular formula of C_30_H_48_.

### 4.2. Phenols

Phenolic compounds are secondary metabolites found in most plants and normally comprise of one or more hydroxyl groups directly attached to one or more aromatic hydrocarbons [143]. Phenols occur in many lower and higher plants, medicinal plants/herbs, and dietary herbs [144], and their distribution is mainly governed by the physiological roles they play within the plant [143,145].

**Figure 9:**
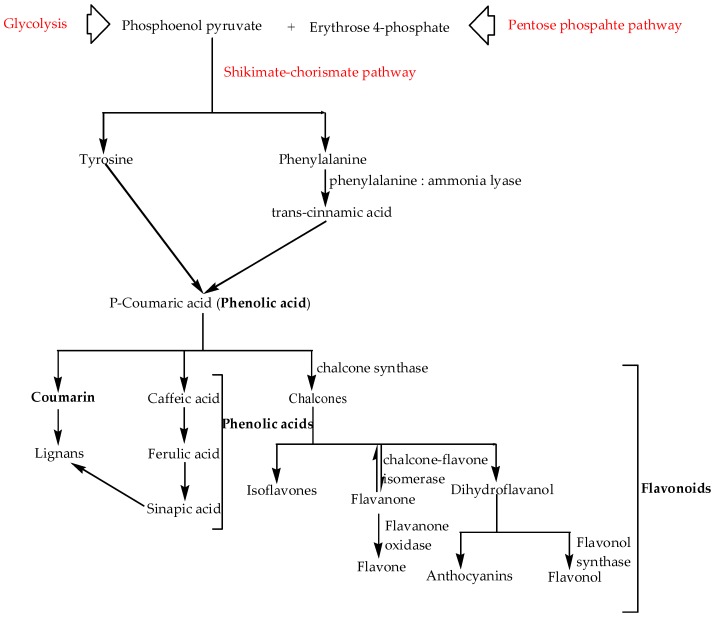
The generalised biosynthetic pathway of phenolic compounds.

There are up to nine (9) groups of compounds classified as phenols, including phenolic acids, phenolic acid analogs, flavonoids, tannins, stilbenes, curcuminoids, coumarins, lignans, and quinones [144,146]. Despite the wide occurrence of phenols in higher plants, only phenolic acids, phenolic acid analogs, flavonoids, tannins, and coumarins have been reported to occur in cotton seeds (41 ppm), bracts (22.6 ppm), leaves (21.6 ppm), and roots [19]. Phenolic compounds are synthesized within the chloroplast of plant cells through a series of reactions which are preceded by the synthesis of aromatic amino acids tyrosine and phenylalanine via the shikimate-chorismate pathway. This pathway involves reactions between phosphoenol pyruvate (a by-product of glycolysis) and erythrose 4-phosphate (a by-product of the oxidative pentose phosphate pathway). These two aromatic amino acids, regarded as the major precursors in the synthesis of phenolic compounds, undergo a series of reactions via the phenylpropanoid pathway resulting in different classes of phenolic compounds (Figure 9). Several other key enzymes are implicated in the synthesis of phenols from one class to another. 

#### 4.2.1. Flavonoids

**Figure 10:**
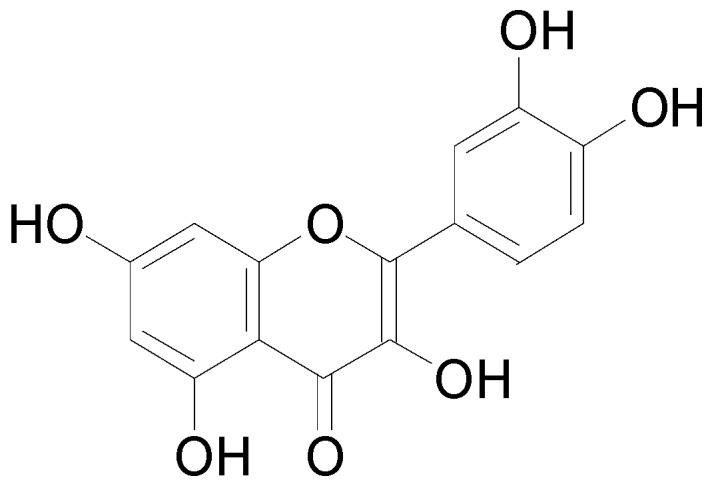
Quercetin, a flavonoid with the basic skeletal structure of flavonoids.

Flavonoids are the most abundant class of phenolic compounds. Huang, Cai and Zhang [144] reported that over 4000 flavonoids occur in nature while Cheynier [143] suggested that the number is closer to 8000. Flavonoids derive their name from the latin word “*flavus*” which means “yellow”, because of the prevalent yellow colour and are largely responsible for the colours of flowers, leaves, barks, fruits, and seeds of most plant species [134]. Flavonoids have a basic skeletal structure of phenyl benzopyrone (C6-C3-C6) comprised of two aromatic rings linked by three carbon atoms. Flavonoids occur as free compounds e.g., quercetin (Figure 10) or as glycosides combined with different sugars [144] e.g., kaempferol 3-glycosides, and quercetin 3-glycosides.

**Figure 11:**
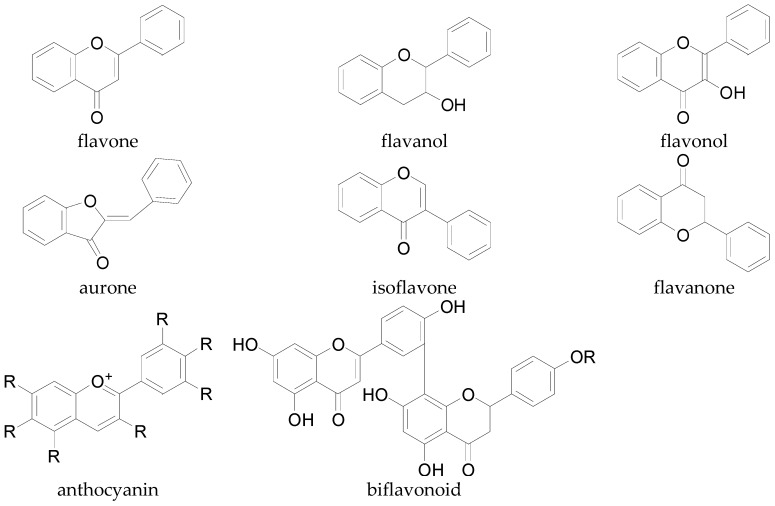
Base structures of the different classes of flavonoids.

**Figure 12:**
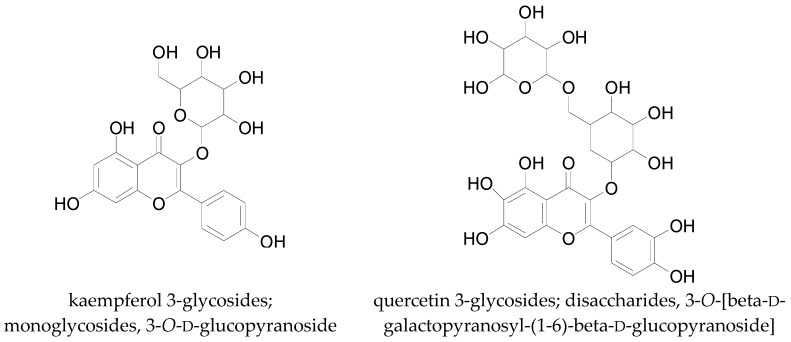
Chemical structures of some flavonoids in cotton.

There are several different classes of flavonoids such as the flavones, flavonols, isoflavones, aurones, anthocyanins, biflavonoids, flavanols, and flavanones [134,147] (Figure 11). These flavonoids differ slightly in their chemical structures. The flavonols possess hydroxyl side groups, which distinguishes them from the flavones. Isoflavones differ from flavones by the location of the phenyl group, whereas the anthocyanins differ from other flavonoids by possessing a positive charge. Biflavonoids have a general formula of (C6-C3-C6)_2_ and aurones possess a chalcone-like group instead of the six-membered ring typical of flavonoids. Several of these flavonoids have been identified in cotton including flavones, and flavonols which mostly occur as glycosides located in flowers, leaves, and seeds [19]. The most common flavonoids in cotton are glycosides of kaempferol, quercetin, and herbacetin (Figure 12). Flavonoid glycosides are water- and ethanol-soluble, while free flavonoids are only soluble in organic solvents [134].

#### 4.2.2. Phenolic Acids and Analogs

**Figure 13:**
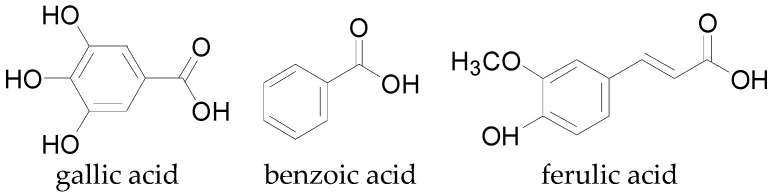
Chemical structures of some phenolic acids present in cotton.

Phenolic acids and their analogs are another group of phenolics that occur in cotton. The hydroxybenzoic acids (HDBA), gallic acid (Figure 13), *p*-hydroxybenzoic acid, protocatechiuc acid, and others listed in Table 1 are common secondary metabolites in cotton, as well as being the predominant phenolic acids in nature. The hydroxycinnamic acids (HDCA) are hydroxyl derivatives of cinnamic acids with a basic C6-C3 structure. Some HDCA identified in cotton plants include chlorogenic acid, ferulic acid (Figure 13), and *p*-coumaric acid which are precursors in the biosynthetic pathway to other phenolic compounds such as the lignins, coumarins, and flavonoids [144].

**Figure 14:**
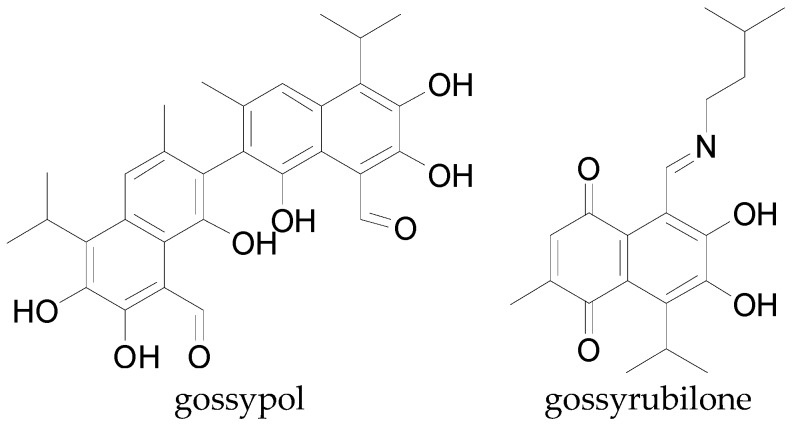
Chemical structures of some phenolic acid analogs present in cotton.

Most phenolic acids have a bitter taste and presumably contribute to the bitter taste of cottonseed products [19]. Gossypol, gossypurpurin, gossyrubilone, and other phenolic acid analogs [30,144] presented in Figure 14 are common secondary compounds isolated from cotton seeds and it is believed they occur in other parts of the cotton plant.

#### 4.2.3. Tannins and Coumarins

**Figure 15:**
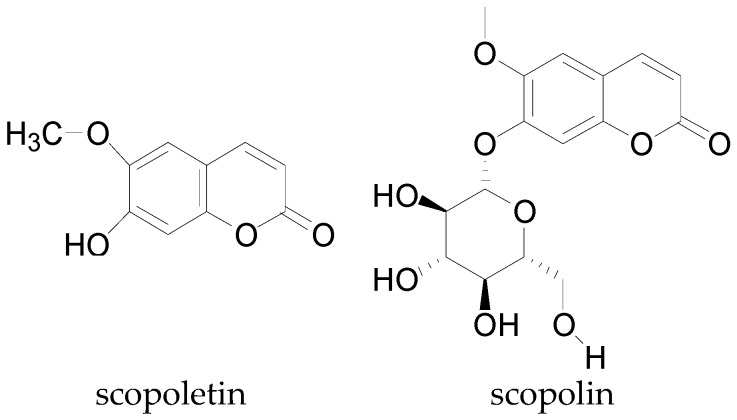
Chemical structures of scopoletin and its glycoside scopolin.

Tannins are a large class of poly phenolic water-soluble compounds which have molecular weights in the range of 500–4000 g/mol. Plant tannins are divided into two classes, the hydrolysable tannins which derive their base unit from gallic acid, and condensed tannins, which arise from proanthocyanidins (condensed flavonols), as well as flavonoid and non-hydrolyzable tannins [144]. Condensed tannins are normally found in combination with alkaloids, polysaccharides, or proteins. These are the class of tannins reported to occur in cotton [148] and act as pesticides, protecting the cotton plant against predators [19]. The coumarins are another group of phenolic acids isolated from cotton. Scopoletin, a coumarin derivative and its glycoside, scopolin presented in Figure 15 have been identified in cotton plant tissue confirming the report that coumarins occur in the free form and as glycosides in cotton, as well as other plants [144].

### 4.3. Fatty Acids, Carbohydrates and Proteins

**Figure 16:**
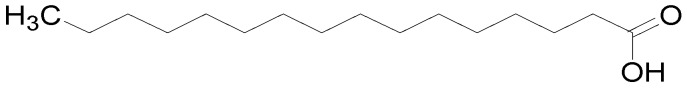
Palmitic acid, a base fatty acid from which other fatty acids are formed.

**Figure 17:**
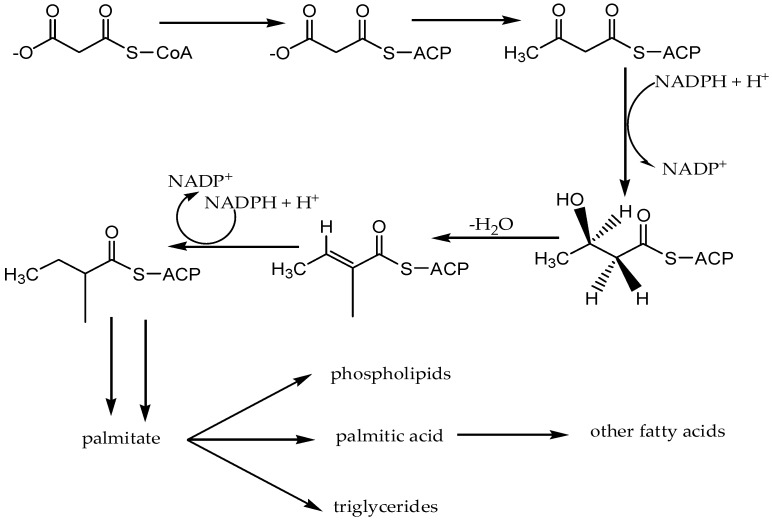
Fatty acid biosynthetic pathway in plants.

Fatty acids are carboxylic acids with long aliphatic chains that are synthesized in the cytosol of plant cells from malonyl-CoA, which in turn is derived from acetyl-CoA. Palmitic acid (Figure 16) is a base fatty acid from which other fatty acids are formed by 2-carbon increments or reduction. The synthesis of palmitic acid (Figure 17) from the precursor malonyl-CoA follows a five step repeating cycle of acylation, condensation, reduction, dehydration, and reduction, which is catalyzed by the fatty acid synthase complex [149,150].

Saturated fatty acids which occur in the cotton plant include myristic acid (tetradecanoic acid), melissic acid (triacontanoic acid), palmitic acid (hexadecanoic acid), stearic acid (octadecanoic acid), and palmitoleic acid (9-hexadecanoic acid) [18,19,151]. Unsaturated fatty acids identified in cotton include eicosadienoic acid, linoleic acid (octadecadienoic acid), linolenic (octadecatrienoic acid), and elaidic acid (octadecenoic acid) [18,19]. Most fatty acids identified in cotton are free fatty acids (not linked to any molecules) and play functional roles as a source of energy for plant growth [19]. 

Cotton, like all plants, is comprised of cellulose and hemicelluloses, proportions of which vary between different parts of the plant. Cotton fibre itself is comprised mainly of cellulose at levels greater than 94% by weight [25]. Raffinose is a unique minor sugar found in cotton plants predominantly in the seed [117,118].

Alkali and water soluble proteins are also found in cotton [152], including water soluble globular proteins vicilin and legumin (Table 1) present in the seeds of cotton [20]. Proline-rich protein H6 is involved in the development of the cell wall structure of cotton fibre [153,154].

### 4.4. Variation in Cotton Chemical Composition 

#### 4.4.1. Genotypes and Varieties

Cotton plants can be categorised as glanded and glandless cotton. Glanded cotton contains pigment glands distributed in tissues and organs of the cotton plant which are rich in gossypol and terpenoid aldehydes [155]. Glandless cotton was developed from the wild-type glanded cotton by McMichael [156] in order to tackle the challenge of gossypol extraction from cottonseed and cotton seed oil [157]. Since then, different varieties of glandless and glanded cotton have been developed, but the absence of pigment glands has made glandless cotton susceptible to infection and pest infestation [155,158]. Glanded cotton contains more proteins, fatty acids, sugars, and terpenoids in comparison with glandless cotton [159], with very little variation between varieties within each group when cultivated under the same environmental conditions [151,160], although Dowd et al. [151] found variation in fatty acid composition was influenced more by genotype than environmental factors, with up to 62.4% of palmitoleic acid content being controlled by genotype and only 5.4% of the variation in linoleic acid induced by environment.

#### 4.4.2. Non-Transgenic and Cotton Transgenic Cotton Differences

Cultivation of transgenic Bt cotton has been widely practised [161,162] which has led to interest in the possibility of induced alterations in the chemical composition, as well as nutritional value of Bt cotton. Yan, et al. [163] reported that all chemical compounds present in non-transgenic cotton were also present in transgenic cotton and indicated that Bt cotton contained higher concentration of some monoterpenes e.g., alpha and beta pinene and lesser concentrations of myrcene and ocimene when compared to non-transgenic cotton. It has been suggested that the increased production of pinene in Bt cotton can be attributed to the activity of genes which cause the plant to repel insects/pests [164,165,166]. Nutritional evaluation of Bt cotton relative to non-transgenic cotton by Mohanta, et al. [167] revealed slight variations in concentration of proteins and carbohydrates in both types of cotton. Overall, these findings suggest transgenic cotton differs slightly from non-transgenic cotton by the general composition of proximate constituents (moisture content, crude fat, and total ash), fibres, minerals, and secondary metabolites.

## 5. Pharmacological Properties of Compounds in Cotton

Several studies have emphasized the importance of plants to the pharmaceutical and medical industry [168,169,170]. Cotton is described as a medicinal plant because of the chemical compounds that have been isolated from it [21,83]. A number of compounds found in cotton play pharmacological roles in nature (Table 2) including anti-microbial, anti-inflammatory, cytotoxic, anti-cancer, and contraceptive roles in both humans and animals. Monoterpenes such as myrcene, pinene, camphene, limonene, and sabinene isolated from cotton possess anti- microbial, anti-inflammatory, anti-cancer, anti-oxidant, and gastro-protective properties [28,171,172].

### 5.1. Anti-Microbial Properties

In vitro and in vivo studies with compounds derived from cotton have found they elicit various effects in most experimental cells and animals. Monoterpenes such as pinene present in the leaves of cotton possess anti-microbial activity against fungi and bacteria. Concentrations as low as 5 µg/mL and 117 µg/mL were reported to have anti-microbial activity towards bacteria and fungi, respectively [173,174]. Only positive enantiomers of the compound induced this effect. The phenolic acid 4-hydroxybenzoic acid which has anti-microbial properties against gram positive and gram-negative bacteria at IC_50_ value of 160 µg/mL [175] is another compound present in the leaves of cotton. The degree of anti-microbial activity of these compounds varies across micro-organisms. This was observed in fungal toxicity assays with 4-hydroxybenzoic acid on *Ganoderma boninense* at concentrations as low as 0.5–2.5 µg/mL [176].

### 5.2. Anti-Inflammatory and Anti-Oxidant Properties

Chemical compounds, such as trans-caryophyllene, caryophyllene oxide, α-humulene, and β-amyrin, are compounds which exert different anti-inflammatory properties. α-Humulene and trans-caryophyllene are reported to prevent chemical-induced paw oedema in rats with 50 mg/kg of both compounds inducing the same anti-inflammatory effects as 0.5 mg/kg of dexamethasone (a steroid anti-inflammatory medication) [32]. At doses of 12 mg/kg and 25 mg/kg body weight of experimental mice, caryophyllene oxide induced anti-inflammatory and analgesic properties almost equivalent to that of an aspirin at a dose of 100 mg/kg body weight of the experimental animals [177]. In humans, studies using peripheral blood mononuclear cells (PMBCs), 1, 2, and 5 µg/mL of β-amyrin promoted the secretion of IL-6 cytokine [178] which is actively involved in pro-inflammatory and anti-inflammatory immune responses. Anti-oxidant properties of β-amyrin and farnesene from “in vitro” studies using human blood cells showed that doses as low as 1 µg/mL [178] and 100 µg/mL [179], respectively, induced anti-oxidant activities in a time-dependent manner.

### 5.3. Cytotoxic and Contraceptive Properties

Cytotoxic activities associated with compounds isolated from cotton are mostly reported in relation to cancer cell lines. α-Bisabolol, a common compound present in cotton possesses the ability to induce apoptosis in malignant carcinoma cell lines without affecting the viability of healthy cells [184]. A dose of 2 µM of α-bisabolol is reported to be effective against cancer cell lines, but an increase in dosage from 50 to 250 µM can induce cytotoxicity in normal cells. Another sesquiterpene, caryophyllene oxide, also exhibits cytotoxic properties against cancer cell lines with a minimum dose of 3.125 µM resulting in reduction in viabilities of the target cells, with this effect more pronounced as the dosage increased [185]. Gossypol is a major compound present in cottonseed oil and other parts of the cotton plant and has been found to have contraceptive properties in mammals. In human males, a concentration of 0.3 mg/kg of body weight can induce azoospermia in a time-dependent manner, whereas in male rats, a concentration of 30 mg/kg will induce the equivalent effect [192]. The contraceptive property of gossypol is not restricted to males alone as a study by Randel, Chase, and Wyse [193] indicated that this compound, if administered at a dose of 40 mg/kg body weight of female mammals, induces abnormal oestrous cycles and reduced pregnancy rates.

## 6. Conclusions

In this review, it has been shown that the whole cotton plant is a reservoir of a wide variety of compounds which have a range of biological functions and exploitable applications. The distribution of compounds in the cotton plant provides knowledge of the chemical content of cotton waste derived from harvesting and cotton ginning operations. Potentially valuable chemical compounds with application in food manufacturing, perfumery, and pharmaceutical industries are found in components of these cotton processing by-products (burr, leaves, crushed seeds, sticks, roots, and flowers of the cotton plant). Gossypol, which is known to have contraceptive properties is not only concentrated in the seeds of cotton, but occurs in the roots and possibly in other parts of the plant. Phenolic compounds and terpenes present in the cotton burr stem, leaves, flowers, stalks, and roots have insecticidal, herbicidal, and phytotoxic properties that could be exploited. This review has highlighted that cotton waste products can be sources of biologically valuable compounds. Special consideration should be given to CGT as a low cost resource because it is centrally stockpiled and collocated with existing infrastructure. Therefore, investigating the occurrence of these chemical compounds in cotton by-products can contribute to recycling and value adding of waste generated from cotton ginning.

## Figures and Tables

**Table 1:** Chemical compounds isolated from cotton (*Gossypium*).

Compounds	Molecular Formula	Molecular Weight (g/mol)	References
Terpenes			
Monoterpenes	C_10_H_16_	136.24	
Camphene			[21]
Limonene			[21,23]
Myrcene			[23]
Ocimene			[21,23]
α-pinene			[23]
β-pinene			[23]
Sabinene			[21,23]
α-Thujene			[21]
Sesquiterpenes	C_15_H_24_	204.35	
α-Bergamotene			[78]
Bisabolene			[78]
1(10),4-Cadinadiene; (6β,7β)-form			[79,80]
Caryophyllene			[78]
Copaene			[81]
Guaiadiene: (4β,5α,7β)-form			[81]
Farnesene			[78]
Humulene			[81,82]
Terpene derivatives			
α and β-Amyrin	C_30_H_50_O	426.72	[83]
Bisabolol	C_15_H_26_O	222.37	[82,84]
1,3,5,10-Bisabolatetraen-7-ol	C_15_H_22_O	218.34	[82]
Bisabolene oxide	C_15_H_24_O	220.35	[85]
1(10),4-Cadinadien-2-ol; (2ξ,6β,7β)-form	C_15_H_24_O	220.35	[86]
1,3,5,7,9-Cadinapentaene-3,9-diol	C_15_H_18_O_2_	230.31	[86]
1,3,5,7,9-Cadinapentaene-3,9-diol; 3-Me ether	C_16_H_20_O_2_	244.33	[86]
1,3,5,9-Cadinatetraene; 7α*H*-form, 3-Hydroxy	C_15_H_20_O	216.32	[87]
1,3,5-Cadinatriene-3,9-diol; (7α,9α,10α)-form, 9-Ketone	C_15_H_20_O_2_	232.32	[88]
1,3,5-Cadinatriene-3,9-diol; (7β,10α)-form, 9-Ketone	C_15_H_20_O_2_	232.32	[88]
1,3,5-Cadinatriene-3,9,10-triol; (7β,9β,10α)-form, 9-*O*-β-d-Glucopyranoside	C_21_H_32_O_8_	412.48	[89]
3(15),6-Caryophylladien-12-ol; (6*E*)-form	C_15_H_24_O	220.35	[90]
3(15),6-Caryophylladien-12-ol; (6*E*)-form, 6α,7β-Epoxide, Ac	C_17_H_26_O_3_	278.39	[90]
Caryophyllene oxide	C_15_H_24_O	220.35	[82]
3,10-Dihydroxy-1,3,5,7-cadinatetraen-9-one	C_15_H_18_O_3_	246.31	[91]
8,9-Dihydroxy-2,5-dioxo-1(6),3,7,9-cadinatetraen-14-al	C_15_H_14_O_5_	274.27	[91]
2,14-Epoxy-1,3,5,7,9-cadinapentaene-8,9-diol	C_15_H_16_O_3_	244.29	[92]
2,14-Epoxy-1,3,5,7,9-cadinapentaene-8,9,12-triol; 15-Hydroxy, 9-*O*-(6-*O*-sulfo-β-d-glucopyranoside)	C_21_H_26_O_13_S	518.50	[93]
Heliocide H_1_	C_25_H_30_O_5_	410.51	[93,94]
Heliocide H_1_; 7-Me ether	C_26_H_32_O_5_	424.54	[95]
Heliocide H_2_	C_25_H_30_O_5_	410.51	[93]
Heliocide H_2_; 3-Me ether	C_26_H_32_O_5_	424.54	[95]
Heliocide H_3_	C_25_H_30_O_5_	410.51	[93]
Heliocide H_3_; 3-Me ether	C_26_H_32_O_5_	424.54	[95]
Heliocide H_4_	C_25_H_30_O_5_	410.51	[93]
Heliocide H_4_; 3-Me ether	C_26_H_32_O_5_	424.54	[95]
β-Sitosterol	C_29_H_50_O	414.71	[83]
Strigol	C_19_H_22_O_6_	346.38	[96,97]
2,3,8,9-Tetrahydroxy-1,3,5,7,9-cadinapentaen-14-al; 3-Me ether	C_16_H_18_O_5_	290.32	[98]
2,3,9-Trihydroxy-1,3,5,7,9-cadinapentaen-14-al; 3-Me ether	C_16_H_18_O_4_	274.32	[99]
2,8,9-Trihydroxy-1,3,5,7,9-cadinapentaen-14-al	C_15_H_16_O_4_	260.29	[100]
2,8,9-Trihydroxy-1,3,5,7,9-cadinapentaen-14-al; 8-Deoxy	C_15_H_16_O_3_	244.29	[100]
2,8,9-Trihydroxy-1,3,5,7,9-cadinapentaen-14-al; 8-Me ether	C_16_H_18_O_4_	274.32	[100]
3,8,9-Trihydroxy-2,5-dioxo-1(6),3,7,9-cadinatetraen-14-al; 3-Me ether	C_16_H_16_O_6_	304.30	[101]
Phytol	C_20_H_40_O	296.54	[18]
Phenols			
Phenolic acids			
Benzoic acid	C_7_H_6_O_2_	122.12	[22]
Chlorogenic acid	C_16_H_18_O_9_	354.31	[22]
Ferrulic acid	C_10_H_10_O_4_	194.18	[22]
Gallic acid	C_7_H_6_O_5_	170.12	[22]
Gentisic acid	C_7_H_6_O_4_	154.12	[22]
P-coumaric acid	C_9_H_8_O_3_	164.16	[22]
4-hydroxybenzoic acid	C_7_H_6_O_3_	138.12	[22]
3,4-Dihydroxybenzoic acid	C_7_H_6_O_4_	154.12	[22]
Syringic acid	C_9_H_10_O_5_	198.17	[22]
Phenolic acid analogs			
Gossypol; (±)-form, 6-Me ether	C_31_H_32_O_8_	532.59	[102]
Gossypol; (±)-form, 6,6′-di-Me ether	C_32_H_34_O_8_	546.62	[102]
Gossypol; (+)-form	C_30_H_30_O_8_	518.56	[83]
Gossypurpurin	C_60_H_56_N_2_O_13_	1013.11	[103]
Gossyrubilone	C_20_H_25_NO_4_	343.42	[95]
Flavonoids			
α-2′,3,3′,4,4′,6-Heptahydroxychalcone; 2′-*O*-d-Glucopyranoside	C_12_H_22_O_13_	482.40	[104]
3,3′,4′,5,7,8-Hexahydroxyflavone; 3-*O*-β-d-Glucopyranoside	C_21_H_20_O_13_	480.38	[19]
Gossypetin 7-glucoside	C_21_H_20_O_13_	480.38	[19]
3,3′,4′,5,7,8-Hexahydroxyflavone; 8-*O*-α-l-Rhamnopyranoside	C_21_H_20_O_12_	464.38	[105]
Kaempferol 3-glycosides; Monoglycosides, 3-*O*-α-d-Glucopyranoside	C_21_H_20_O_11_	448.38	[19]
3,3′,4′,5,7-Pentahydroxyflavan; (2*S*,3*R*)-form	C_15_H_14_O_6_	290.27	[106]
3,3′,4′,5,7-Pentahydroxyflavone; 3′-*O*-β-d-Glucopyranoside	C_21_H_20_O_12_	464.38	[105]
3,4′,5,7,8-Pentahydroxyflavone	C_15_H_10_O_7_	302.24	[107]
Quercetin 3-glycosides; Disaccharides, 3-*O*-[β-d-Galactopyranosyl-(1→6)-β-d-glucopyranoside]	C_27_H_30_O_17_	626.52	[105]
Quercetin 3-glycosides; Tetra- and higher saccharides, 3-*O*-[α-d-Apiofuranosyl-(1→5)-β-d-apiofuranosyl-(1→2)-[α-l-rhamnopyranosyl-(1→6)]-β-d-glucopyranoside]	C_37_H_46_O_24_	874.76	[108]
3,3′,5,7-Tetrahydroxy-4′-methoxyflavone	C_16_H_12_O_7_	316.27	[19]
3,4′,5,7-Tetrahydroxy-8-methoxyflavone; 3-*O*-β-d-Glucopyranoside, 7-*O*-α-l-rhamnopyranoside	C_28_H_32_O_16_	624.55	[109]
Other Phenols			
Scopoletin	C_10_H_8_O_4_	192.17	[110]
Fatty acids and Lipids			
11,14-Eicosadienoic acid	C_20_H_36_O_2_	308.50	[24]
Hexadecanoic acid	C_16_H_32_O_2_	256.43	[24]
9-Hexadecanoic acid; (*Z*)-form	C_16_H_30_O_2_	254.41	
Octadecanoic acid	C_18_H_36_O_2_	284.48	[24]
9-Octadecenoic acid; (*Z*)-form	C_18_H_34_O_2_	282.47	[24]
9,12-Octadecadienoic acid; (*Z*,*Z*)-form	C_18_H_32_O_2_	280.45	[18]
9,12,15-Octadecatrienoic acid; (*Z*,*Z*,*Z*)-form	C_18_H_30_O_2_	278.43	[18]
Tetradecanoic acid (myristic acid)	C_14_H_28_O_2_	228.37	[24]
Triacontanoic acid	C_30_H_60_O_2_	452.80	[111]
Carbohydrates			
Cellulose	C_6_H_10_O_5_	162.14	[25]
Cyanidin 3-glycosides; Disaccharides, 3-*O*-[β-d-Xylopyranosyl-(1→4)-β-d-glucopyranoside]	C_26_H_29_O_15_	581.51	[112]
6-*O*-α-d-Galactopyranosyl-d-glucose	C_12_H_22_O_11_	342.30	[113,114]
Glycerol 1-alkanoates; Glycerol 1-(22-hydroxydocosanoate), 22′-*O*-(3,4-Dihydroxycinnamoyl)	C_34_H_56_O_8_	592.81	[115,116]
Raffinose	C_18_H_32_O_16_	504.44	[117,118]
Proteins			
3-Phosphoglycerate phosphatase			[119]
Vicilin A and B			[20]
Legumin Aand B			[20]
Hydrocarbons			
1*H*-Indole-3-carboxaldehyde	C_12_H_7_NO	145.16	[120,121]
1-Methyl-2-propylbenzene	C_10_H_14_	134.22	[122]
Octatriacontane	C_38_H_78_	535.03	[123]
Alcohols			
Dotriacontanol	C_32_H_66_O	466.88	[18]
1-Tetratriacontanol	C_34_H_70_O	494.93	[18]
Triacontanol	C_30_H_62_O	438.81	[18]

**Table 2:** Biological activities of different compounds present in cotton.

Compounds	Biological Activity	References
Terpenes		
camphene	Aromatic properties, antioxidants effects	[21]
limonene	Flavouring properties. gastro-protective effects, anti-cancer and anti-inflammatory activity	[28,142]
myrcene	Analgesic effects, anti-microbial activity, anti-inflammatory activity, anti-catabolic activity	[171,172]
α and β-pinene	Gastro-protective effects, anti- microbial and ant-inflammatory effects	[28,174,180]
sabinene	Anti-microbial activity, anti-oxidant activity	[21]
α-thujene	Pungent activity	[21]
caryophyllene	Ant-inflammatory effects, anti-microbial activity, regulation of cellular lipid metabolism, flavouring properties	[27,32,181,182]
farnesene	Anti-oxidant effects	[179]
humulene	Anti-inflammatory properties, aromatic properties and cytotoxic activity	[29,32]
bisabolol	Aromatic properties, anti-inflammatory effects, anti-carcinogenic activity, anti-microbial and anti-oxidative properties	[28,183,184]
caryophyllene oxide	Cytotoxic activity, phytogrowth inhibition, analgesic and anti-inflammatory activity	[177] [26,185]
3,10-dihydroxy-1,3,5,7-cadinatetraen-9-one	Phytoalexin, antifungal agent	[92,186]
β-sitosterol	Antimicrobial activity, anti-hypercholesteraemic and anti-inflammatory activity	[18,28]
strigol	Germination stimulant	[96,187,188]
2,3,9-trihydroxy-1,3,5,7,9-cadinapentaen-14-al; 3-Me ether	Phytoalexin	[99]
2,8,9-trihydroxy-1,3,5,7,9-cadinapentaen-14-al; 8-deoxy	Antifungal activity	[92]
Phenols		
chlorogenic acid	Anti-oxidant and anti-mutagenic activity	[144]
gallic acid	Antioxidant activity, cytotoxic activity	[189,190]
4-hydroxybenzoic acid	Anti-microbial activity, used as preservative, oestrogenic activity, anti-inflammatory and anti-oxidant activity	[175,176,191]
gossypol; (+)-form	Contraceptive and hypokalemic activity	[30,192,193]
3,3′,4′,5,7-pentahydroxyflavan; (2*S*,3*R*)-form	Cytotoxic and phytotoxic activity	[194,195]
3,3′,4′,5,7-pentahydroxyflavone; 3′-*O*-β-d-glucopyranoside	Enzyme inhibitor, cytotoxic, anti-oxidant activity	[196]
scopoletin	Anti-spasmodic and anti-inflammatory activity	[19]
Fatty acids		
11,14-eicosadienoic acid	Hormonal activity	[18]
hexadecanoic acid	Anti-microbial and anti-inflammatory activity	[197]
octadecanoic acid	Pharmaceutical excipient, surfactant and softening activity	[198]
9-octadecenoic acid; (*Z*)-form	Insecticidal, anti-bacterial and fungicidal activity	[199,200,201]
tetradecanoic acid	Defoaming agent, flavour adjuvant used in food processing	[202]
Carbohydrates		
cellulose	Capsule and tablet diluent	[203]
Proteins		
3-phosphoglycerate phosphatase	Enzyme activity	[119]
vicilin	Anti-hypertensive activity	[204]
